# The Predictive Value of ALBI Score for No-Reflow in Non-ST Elevation Acute Coronary Syndrome

**DOI:** 10.3390/jcm14093035

**Published:** 2025-04-28

**Authors:** Abdullah Yildirim, Mukremin Coskun, Abdullah Orhan Demirtas

**Affiliations:** Department of Cardiology, University of Health Sciences, Adana City Training and Research Hospital, 01230 Adana, Türkiye; mukremincoskun@gmail.com (M.C.); aorhandemirtas@gmail.com (A.O.D.)

**Keywords:** albumin-bilirubin (ALBI) score, no-reflow phenomenon, non-st elevation acute coronary syndrome, percutaneous coronary intervention, machine learning (XGBoost; SHAP)

## Abstract

**Background:** The albumin–bilirubin (ALBI) score, initially a hepatic function marker, may also reflect systemic inflammation and oxidative stress, both linked to the no-reflow phenomenon (NRP). This study investigates the ALBI score’s predictive value for the NRP and compares it with conventional risk models. **Methods:** This retrospective, single-center study included 1563 NSTE-ACS patients who underwent PCI between January 2023 and February 2024. Two predictive models were developed: (i) a fitted model with variables selected based on the XGBoost algorithm and SHapley Additive ExPlanations (SHAP) values, and (ii) an ALBI model including the ALBI score. Machine learning via the XGBoost algorithm was used for modeling, with SHAP applied to assess the significance of predictors. **Results:** The NRP occurred in 14.8% (231/1563) of patients. The ALBI score emerged as an independent predictor (OR = 12.10, 95% CI: 7.75–18.89, *p* < 0.001). The ALBI model demonstrated superior predictive power compared to the fitted model (C-index: 0.860 vs. 0.799), with significant improvements in discrimination (11.1%, *p* < 0.001) and reclassification (14.5%, *p* = 0.002). SHAP analysis ranked the ALBI score (1.025) as the strongest predictor, followed by hs-TnI (0.814), e-GFR (0.582), and pre-dilatation (0.283). The ALBI model exhibited better specificity (AUC: 0.860 vs. 0.798), calibration (Brier score: 0.088 vs. 0.102), and model fit (AIC: 964.7 vs. 1098.3) compared to the fitted model, indicating superior overall performance. **Conclusions:** The ALBI score significantly enhances the prediction of the NRP in NSTE-ACS patients undergoing PCI, outperforming traditional risk models. Incorporating the ALBI score into predictive frameworks may improve early risk stratification and guide clinical decision-making.

## 1. Introduction

In-hospital events such as bleeding and arrhythmias, as well as procedural complications, are still important determinants of outcomes in acute coronary syndrome (ACS) [1,2]. Among these, the no-reflow phenomenon (NRP) is defined as the continued inadequacy of myocardial perfusion despite the successful mechanical reopening of the infarct-related artery following percutaneous coronary intervention (PCI) [2,3]. Although the mechanisms underlying the NRP are not entirely clear, distal thrombus embolization, microvascular obstruction, and coronary spasm are considered the primary causes [4,5]. The incomplete restoration of flow results in insufficient perfusion, significantly diminishing the benefits of PCI and exacerbating myocardial tissue damage [3]. Consequently, this leads to an increased incidence of heart failure, malignant arrhythmias, cardiogenic shock, and in-hospital mortality [2,6,7].

The albumin–bilirubin (ALBI) score is a contemporary parameter calculated using serum albumin and plasma total bilirubin levels, initially utilized to assess the hepatic reserve capacity in patients with liver disease [8]. However, recent studies have demonstrated that the application of the ALBI score is not confined to the gastrointestinal system; it has shown similar potential in heart failure [9], cardiomyopathies [10], congenital heart diseases [11], and patients undergoing valve intervention [12]. In our study, we investigated whether the ALBI score is an important clinical factor for predicting the NRP after PCI in patients with non-ST elevation ACS (NSTE-ACS).

## 2. Materials and Methods

### 2.1. Study Design and Setting

**Figure 1:**
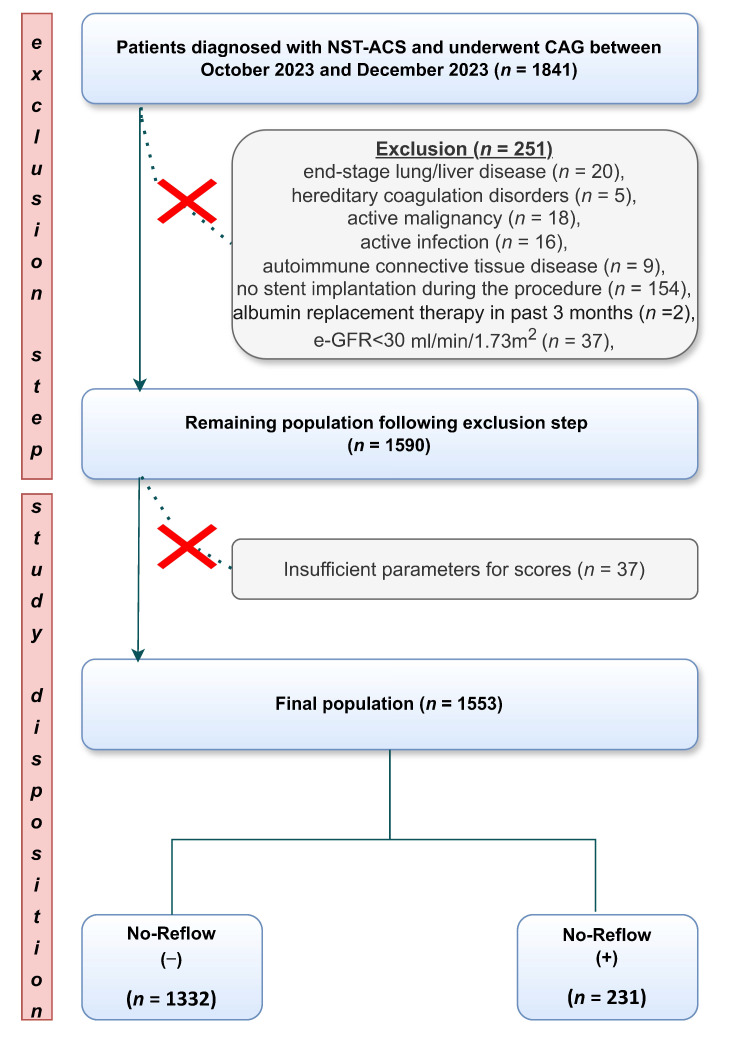
The flowchart illustrates the selection process of patients diagnosed with NST-ACS who underwent percutaneous coronary intervention.

This retrospective single-center study was conducted between October 2023 and December 2023. A total of 1853 patients diagnosed with NSTE-ACS and undergoing coronary angiography were initially screened. After applying the following exclusion criteria—end-stage lung or liver disease (*n* = 20), hereditary coagulation disorders (*n* = 5), active malignancy (*n* = 18), active infection (*n* = 16), autoimmune connective tissue disease (*n* = 9), no stent implantation during the procedure (*n* = 154), albumin replacement therapy within the past 3 months (*n* = 2), and estimated glomerular filtration rate (e-GFR) < 30 mL/min/1.73 m^2^ (*n* = 37)—251 patients were excluded. An additional 39 patients were excluded due to insufficient data required for the ALBI score calculation. Finally, 1553 patients who underwent PCI were included in the analysis (Figure 1). The diagnosis of NSTE-ACS was established according to the 2023 European Society of Cardiology (ESC) guidelines, using the 0/2 h algorithm for high-sensitivity cardiac troponin I (hs-cTnI) in line with clinical practice [13]. A 12-lead ECG was obtained from all patients upon admission. Each patient’s medical history, comorbidities, and medication data were retrospectively obtained from national health registry systems, health records, and patient interviews. Hypertension was defined as systolic blood pressure ≥ 140 mm Hg and/or diastolic blood pressure ≥ 90 mm Hg or the current use of antihypertensive medications.

### 2.2. Laboratory Analysis and ALBI Score Calculation

Laboratory analyses were performed using antecubital venous blood samples collected at the time of admission. The blood samples were analyzed with the fully automated chemistry analyzer Beckman UniCel DXC 800 Synchron (Beckman Coulter Inc., Brea, CA, USA). Serum CK-MB and hs-cTnI levels were measured using the chemiluminescence method with a UniCel DXI 800 Synchron (Beckman Coulter Inc., Brea, CA, USA) autoanalyzer. The e-GFR was calculated according to the Modification of Diet in Renal Disease (MDRD) formula [14]. Patients with chronic kidney disease were defined as those with stage 3b (e-GFR < 30 mL/min/1.73 m^2^) or the presence of known ongoing renal disease (e.g., renal replacement therapy, history of renal transplantation, proteinuria, or albuminuria) and were excluded. The ALBI score was calculated based on baseline serum albumin and total bilirubin levels using the following formula: (log_10_ bilirubin [mmol/L] × 0.66) − (albumin [g/L] × 0.085) [8].

### 2.3. Coronary Angiography

All patients underwent coronary angiography using a SIEMENS AXIOM Artis system (Siemens Healthcare, Erlangen, Germany) with 5–7 French catheters via either femoral or radial access. Patients received acetylsalicylic acid (300 mg loading dose, followed by a 100 mg daily maintenance dose) and a P2Y12 inhibitor—either ticagrelor (180 mg loading dose, followed by 90 mg twice daily) or clopidogrel (600 mg loading dose, followed by 75 mg daily)—in line with the current guidelines. Following the decision for coronary intervention, unfractionated heparin (70–100 IU/kg bolus, with an additional 30–50 IU/kg for extended procedures) was administered. The choice of the guiding catheter type, stent size, decision on and size of pre- and post-dilatation balloons, thrombus aspiration, and glycoprotein IIb/IIIa inhibitor infusion (tirofiban, given as a 25 µg/kg bolus followed by a continuous 12 h infusion at 0.15 µg/kg/min) were all left to the operator’s discretion. Moreover, high-dose statins (atorvastatin 40–80 mg or rosuvastatin 20–40 mg), beta-blockers, and ACE inhibitors/ARBs were initiated in eligible patients, adhering to current guidelines and considering contraindications.

Transthoracic echocardiographic assessments were performed by experienced cardiologists during hospitalization. The left ventricular ejection fraction was calculated using the modified Simpson method. Coronary angiograms were independently evaluated by two interventional cardiologists using a digital DICOM viewer (MedCom GmbH, Darmstadt, Germany). Coronary flow was assessed according to the thrombolysis in myocardial infarction (TIMI) flow classification (grade 0, 1, 2, or 3). A TIMI flow grade of 2 or lower, in the absence of mechanical obstruction, spasm, and significant stenosis, was considered the NRP [15,16]. The TIMI thrombus scale (grade 0 [no thrombus], 1, 2, 3, 4, and 5 [large thrombus causing total occlusion]) was utilized to evaluate the thrombus burden [17]. The GRACE score for each patient was calculated based on variables including age, heart rate, systolic blood pressure, creatinine level, Killip class, cardiac arrest at admission, elevated cardiac markers, and ST-segment deviations.

### 2.4. Statistical Preparation

Data and statistical analyses for this study were performed using the R Foundation for Statistical Computing (version 4.4.2, Vienna, Austria; URL: http://www.r-project.org). Categorical variables were presented as counts (percentages). The distribution of continuous variables was assessed using visual tests, such as histograms, skewness, and kurtosis, in addition to the Kolmogorov–Smirnov test, to identify normally distributed variables. Normally distributed variables were expressed as the mean ± standard deviation. Continuous variables were analyzed using the independent sample *t*-test, while categorical variables were compared using the χ^2^ test or Fisher’s exact test, as appropriate. The importance of the variables in the model was evaluated using SHapley Additive exPlanations (SHAP) values based on the XGBoost machine learning algorithm [18].

To evaluate the additional prognostic role of the ALBI score, two models were constructed: a “fitted model” using the prognostic variables, and an “ALBI model” obtained by adding the ALBI score to the fitted model. Model calibration was visually represented through calibration plots, contrasting observed outcomes on the y-axis with predicted outcomes on the x-axis. In all regression analyses, odds ratios (ORs), adjusted-ORs (a-ORs), and 95% confidence intervals (CIs) were calculated. Multicollinearity was examined using the variance inflation factor, with values greater than 3 indicating significant multicollinearity. The Hosmer–Lemeshow test was used to evaluate the goodness-of-fit of logistic regressions. Model performance was evaluated using several metrics: Akaike Information Criterion (lower values indicating better fit), Brier score (lower values indicating better calibration), adjusted R^2^ (higher values indicating better fit), area under the curve (AUC < 0.50 indicates no discrimination, >0.75 indicates good discrimination), and the C-statistic (higher values indicate better discrimination). The predictive performance of the ALBI score for NRP detection was evaluated using a k-fold cross-validation approach. In this method, the dataset was divided into eight subsets, with the model being iteratively trained on four folds and tested on the remaining fold. Receiver operating characteristic (ROC) curve analysis was conducted for each fold, with corresponding AUC values calculated. The overall performance was represented by the average k-fold AUC along with its 95% CI. The additional prognostic value of the ALBI model over the fitted model was assessed using Integrated Discrimination Improvement (IDI), continuous Net Reclassification Improvement (NRI), and median improvement analyses [19]. Finally, Decision Curve Analysis (DCA) was utilized to compare the net clinical benefit between the ALBI and fitted models. The net benefit in predicting the NRP was defined such that a value of zero indicated no net benefit, while values greater than zero indicated an increasing net benefit. All statistical analyses used two-sided tests with a significance level (alpha) of 0.05.

## 3. Results

### 3.1. Baseline Characreristics, Procedural Data, and Outcomes

A total of 1563 patients (mean age: 62.3 ± 10.9 years; 46.9% female) were enrolled and categorized based on the presence of the NRP into two groups: NRP (−) [*n* = 1332, 85.2%] and NRP (+) [*n* = 231, 14.8%]. The diastolic blood pressure, heart rate, and left ventricular ejection fraction were lower in the NRP (+) group (*p* = 0.004, *p* < 0.001, and *p* = 0.009, respectively). Apart from differences in diabetes mellitus, the history of PCI, the smoking status, and chronic heart failure (*p* = 0.024, *p* < 0.001, *p* < 0.001, and *p* < 0.001, respectively), all other demographic and baseline characteristics were comparable between the groups. Laboratory parameters including creatinine, e-GFR, hs-CRP, albumin, bilirubin, and hs-cTnI levels were significantly different between the groups (all *p* < 0.001). Furthermore, hemoglobin levels were significantly lower in the NRP (+) group (*p* < 0.001). At admission, ACE inhibitor/ARB and statin usage was significantly higher, while beta-blocker usage was lower in the NRP (+) group (all *p* < 0.05) (Table 1). In terms of angiographic characteristics, the distribution of culprit lesions and thrombus burden was similar between the two groups. However, glycoprotein IIb/IIIa inhibitor infusion, inotrope usage, shock, malignant arrhythmias, multi-vessel disease, and in-hospital mortality were significantly more frequent in the NRP (+) group (all *p* < 0.05) (Table 2).

### 3.2. Feature Selection and Modeling with the XGBoost Machine Learning Algorithm

**Figure 2:**
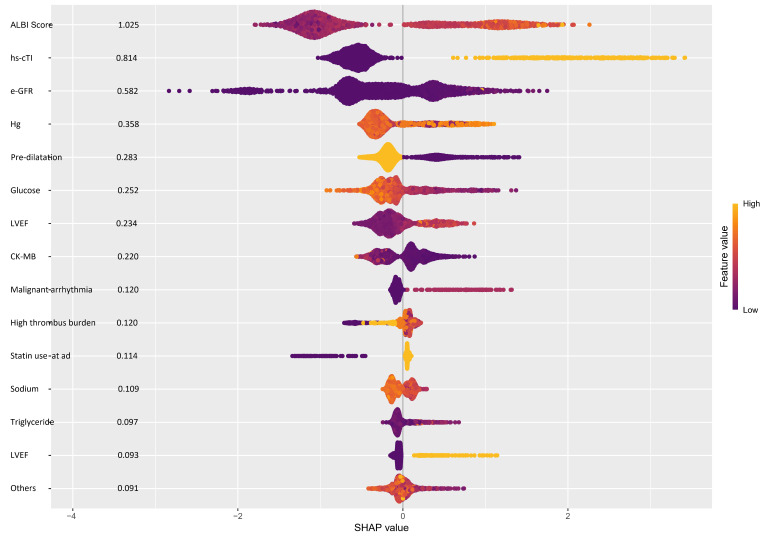
SHAP summary plot illustrating the relative importance of features in predicting the occurrence of no-reflow using the XGBoost machine learning algorithm.

Among the parameters constituting the ALBI score model, the ALBI score itself provided the highest contribution (SHAP value = 1.025), followed by hs-cTnI (0.814), e-GFR (0.582), the hemoglobin level (0.358), pre-dilatation (0.283), and the blood glucose level (0.252) (Figure 2).

**Figure 3:**
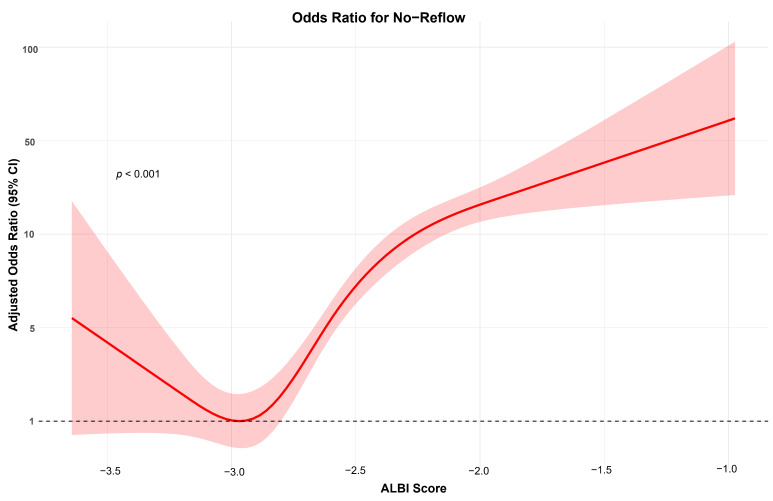
The effect of the ALBI score on admission, treated as a continuous variable, on the occurrence of no-reflow. The baseline (red line) is adjusted for key covariates, including hs-cTnI, e-GFR, the hemoglobin level, pre-dilatation, the glucose level, and other significant parameters identified based on SHAP values. The red shaded area represents the 95% confidence interval, indicating the range of uncertainty around the estimated effect. The black dotted line represents an odds ratio of 1 (no effect).

The multivariable logistic regression analyses, conducted separately for the covariate effect, the fitted model, and the ALBI score model using the variables derived from the XGBoost algorithm, revealed the following associations. In the ALBI score model, the following variables were independently associated with the NRP: hs-cTnI (OR = 1.00, 95% CI:1.00–1.00, *p* < 0.001), pre-dilatation (OR = 0.24, 95% CI:0.14–0.40, *p* < 0.001), hemoglobin level (OR = 0.84, 95% CI:0.77–0.93, *p* < 0.001), CK-MB level (OR = 0.99, 95% CI:0.99–1.00, *p* = 0.043), malignant arrhythmia (OR = 4.61, 95% CI:2.81–7.56, *p* < 0.001), statin use at admission (OR = 4.10, 95% CI:2.51–6.71, *p* < 0.001), and ALBI score (OR = 12.10, 95% CI:7.75–18.89, *p* < 0.001) (Table 3 and Figure 3).

### 3.3. Discrimination, Calibration, and Performance Evaluation of the XGBoost Model

**Figure 4:**
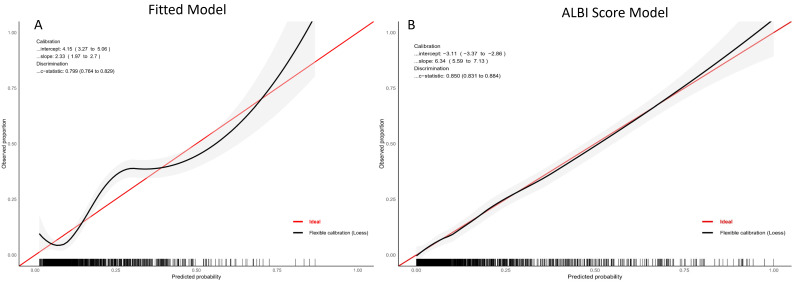
Calibration plots illustrating the prognostic performance of the fitted model and the ALBI score model in predicting no-reflow. Calibration plots for the (**A**) fitted model and (**B**) ALBI score model.

**Figure 5:**
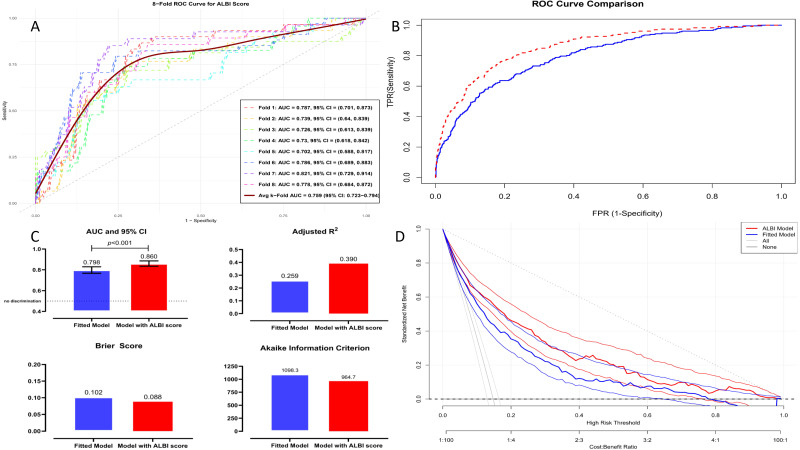
(**A**) The plot displays ROC curves for each fold in a k-fold cross-validation, showing the ALBI score performance in predicting no-reflow. The average 8-fold AUC with a 95% CI is also included for the overall evaluation. (**B**) ROC curves comparing the discriminative performance of the fitted model (blue line) and the ALBI score model (red line) in predicting no-reflow. (**C**) Insets display key performance metrics, including the AUC, adjusted R^2^, Brier score, and AIC, providing a comparative evaluation of the two models’ predictive accuracy and calibration. (**D**) Decision curve analysis displaying the standardized net benefit of the ALBI score model and the fitted model across a range of high-risk thresholds. The curves for treating all patients (gray line) and treating none (black line) are also included for comparison. Abbreviations: AIC, Akaike Information Criterion; AUC, area under the curve; CI, confidence interval; ROC, receiver operating characteristic.

The ALBI score model yielded higher discrimination for the NRP, with a C-index of 0.860 (95% CI:0.831–0.884, *p* < 0.001) compared to the fitted model (C-index = 0.799, 95% CI:0.764–0.829, *p* < 0.001) (Figure 4A,B). Internal validation using k-fold cross-validated ROC curves revealed an average AUC of 0.759 (95% CI: 0.723–0.794) for the ALBI model, indicating moderate discriminative power across the folds (Figure 5A). Furthermore, the ALBI model exhibited significant improvements over the fitted model in terms of reclassification (14.5%, *p* = 0.002), the discrimination index (11.1%, *p* < 0.001), and median improvement (10.5%, *p* < 0.001) (Table 4).

In the ROC curve comparison, the ALBI model demonstrated superior specificity with an AUC of 0.860 (95% CI: 0.834–0.886, *p* < 0.001), compared to the fitted model’s AUC of 0.798 (95% CI: 0.767–0.829, *p* < 0.001) (Figure 5B). Moreover, the ALBI model consistently outperformed the fitted model in key statistical measures, including the Akaike Information Criterion (964.7 vs. 1098.3), adjusted R^2^ (0.390 vs. 0.259), and the Brier score (0.088 vs. 0.102) (Figure 5C). Finally, the ALBI model consistently provided a better net clinical benefit compared to the fitted model, as shown in the decision curve analysis (Figure 5D).

## 4. Discussion

The main findings of the present study were as follows: (*i*) the NRP was observed in 14.8% of NSTE-ACS patients who underwent PCI; (*ii*) the participants were predominantly elderly and male; (*iii*) the ALBI score, hs-cTnI, CK-MB, pre-dilatation, and hemoglobin levels were independent predictors of the NRP in NSTE-ACS patients undergoing PCI; and (*iv*) the ALBI score can enhance the predictive significance of the NRP, with a 14.5% improvement in reclassification and an 11.1% increase in the discrimination index over pre-existing risk factors. Furthermore, the ALBI score showed an additive prognostic value over these established risk factors. Thus, the study results suggest that the ALBI score (a liver dysfunction score) is independent of the risk of the NRP in NSTE-ACS patients who underwent PCI and is capable of providing a better prognostic prediction than that offered by pre-existing risk factors. Moreover, we employed the XGBoost machine learning algorithm rather than conventional statistical analysis techniques to assess the predictive and discriminative power of the ALBI score.

The NRP is an important complication in ACS patients that limits the benefits of PCI. Its incidence has been reported to vary between 11% and 41% in different series and has been associated with in-hospital events, periprocedural myocardial injury, malignant arrhythmias, major adverse cardiovascular events, and increased mortality [2,6,7,20,21,22]. Although the underlying pathophysiological mechanisms are multifactorial, they have not been completely elucidated [23,24]. Inflammation, endothelial dysfunction, and reactive oxygen species are considered the most likely causes [5,23]. Therefore, research has focused on protective measures and practical predictive markers or models [24]. In line with this aim, our study concentrated on the role of the ALBI score, a novel parameter derived from albumin and bilirubin, in relation to the mechanisms of the NRP and its predictive role in forecasting the NRP.

Albumin is an important plasma protein synthesized by the liver. In addition to being a negative acute-phase reactant, it acts as an anti-aggregant by increasing the production of prostaglandins [25]. Moreover, due to its roles in redox-signaling processes regulating inflammation, pH buffering, and the transport of physiologically active substances, albumin possesses anti-inflammatory and antioxidant properties [26]. The role of albumin levels in cardiovascular disease has been frequently investigated [27]. Hypoalbuminemia is closely associated with adverse cardiovascular outcomes in patients with chronic coronary syndrome [28] and plays an important predictive role in the frailty of most cardiovascular diseases, particularly in transcatheter procedures. Recent studies have shown that low albumin levels are associated with the NRP in ST-elevation myocardial infarction (STEMI) patients. For example, Cinar et al. reported a relationship between albumin levels and NRP in STEMI [29]. Similarly, Karabag et al. found, in a study of 1217 STEMI patients, that albumin levels, as well as a high thrombus burden, hemoglobin, and glucose levels, were closely associated with the NRP [30]. Another study reported that low albumin levels in STEMI patients are associated with high in-hospital mortality [31]. This may be explained by the fact that the hypercoagulable state in the coronary microvasculature and reperfusion injury, both of which may contribute to the NRP, are closely linked to decreased albumin levels.

Recent studies have increased the understanding of oxidant–antioxidant pathways and their effects on atherosclerotic processes. Serdar et al. reported a relationship between the severity of ACS and the oxidative status [32]. Another study noted that a disrupted oxidative balance is closely related to endothelial damage, which is important for vascular function [33]. The loss of endothelial function leads to an increase in adhesion molecules, oxidized lipid accumulation in damaged arterial walls, and leukocyte aggregation, all of which are closely associated with accelerated atherosclerotic processes [32,33,34,35]. These processes, coupled with the loss of antithrombotic factors, impaired vascular tone and blood flow, and chemically induced platelet activation, lead to the formation of atherothrombosis [4,33,36,37]. Consequently, the loss of antioxidant function contributes not only to the development of ACS but also to the underlying pathophysiological mechanisms of the NRP, which are closely linked to adverse cardiac outcomes. This also helps to explain the prognostic relevance of inflammation-based scores in predicting the occurrence of the NRP [21,24,29,30,38]. Numerous studies have demonstrated that bilirubin, like albumin, acts as an antioxidant molecule that plays an important role in preventing the oxidation of LDL-C, a key factor in atherosclerosis [39,40]. Elevated bilirubin levels have also been shown to be closely related to the levels of heme oxygenase-1 (HO-1), a stress-related enzyme, in patients with acute myocardial infarction (AMI) [41]. The HO-1 protein, released in the myocardium after AMI, mitigates free oxygen radicals and serves as a protective mechanism in ventricular remodeling [42]. Another study demonstrated the protective role of HO-1 activity in vascular events [43]. Therefore, elevated serum bilirubin levels, which indicate increased HO-1 activity and its antioxidant properties, might protect against vascular events, such as ACS. However, Celik et al. reported that increased serum bilirubin levels are associated with impaired flow after PCI and in-hospital adverse cardiac events [44]. Other studies have indicated that high serum bilirubin levels in STEMI patients are associated with an increased thrombus burden and the extent of coronary artery disease [45,46]. In our study, we demonstrated that the ALBI score and its component, bilirubin levels, are closely related to TIMI flow after PCI in NSTE-ACS patients. These findings may be due not only to the multifactorial causes of elevated bilirubin levels but also to the relationship between the ALBI score, which reflects liver function, and cardiovascular health (cardio-hepatic crosstalk). Decreased liver perfusion and subtle, acquired liver dysfunction may explain the elevated bilirubin levels and low albumin levels observed [47]. Moreover, anemia resulting from hemolysis, which can lead to hyperbilirubinemia, has also been closely associated with the NRP. This relationship has been frequently reported in previous studies [6,30,38,48]. Our study similarly found that low hemoglobin levels were closely associated with the NRP.

The relationship between procedural techniques during coronary angiography and the NRP has been examined in previous studies. One such comparison is between direct stenting and conventional stenting following balloon pre-dilatation. Harbalioglu et al. reported that stenting after pre-dilatation in STEMI patients was associated with the NRP [6]. Although similar results have been reported in other studies, this issue remains controversial. Gasior et al. found no difference between these two stenting strategies in terms of NRP development, although direct stenting was more closely associated with adverse events such as one-year restenosis in the context of stent optimization [49]. In our study, direct stenting was more closely associated with the NRP. These differences may be related to technical variations in PCI procedures, the thrombus burden, and complexity of the lesion, as well as patient-related or operator-dependent factors, such as the hemodynamic status and ischemic time. Since the decision to perform pre-dilatation was at the discretion of the operators, differences between studies might be due to variations in patient conditions across the two stenting strategies.

The major limitation of our study is its single-center and retrospective design, which inherently includes the possibility of hidden or selection bias and may not fully reflect causality. Additionally, inter-operator variability was not formally assessed, although all procedures were performed in a single center under standardized institutional protocols. Although internal validation was performed using cross-validation, the possibility of overfitting cannot be entirely ruled out due to the model’s complexity. Moreover, mid- and long-term follow-up, including major cardiac outcomes such as mortality, were not provided. Our findings do not extend to other subgroups, such as STEMI or chronic coronary syndrome. Despite these important limitations, we aimed to demonstrate the relationship between the ALBI score as a contemporary liver reserve score based on albumin and bilirubin levels and the NRP, as well as the combined role of these two parameters. We found that assessing the independent effect of the ALBI score on the NRP in addition to known risk factors and inflammatory markers, using robust machine learning-based statistical methods in a sufficiently large cohort of NST-ACS patients, is beneficial.

## 5. Conclusions

In individuals undergoing PCI for NST-ACS, the laboratory biomarker-based ALBI score may be associated with the post-PCI NRP. Furthermore, the ALBI score may have better discriminatory power than pre-existing risk factors in this population. However, it is evident that prospective, randomized-controlled trials are needed to externally validate these results.

## Figures and Tables

**Table 1:** Demographic, admission clinical, and laboratory parameters of the overall population.

Variable		Pooled (*n* = 1563)	No-Reflow (−) (*n* = 1332)	No-Reflow (+) (*n* = 231)	*p*-Value *
**Basic characteristics and admission parameters**					
Age, years		62.3 ± 10.9	61.9 ± 10.8	65.2 ± 10.8	<0.001
Gender, male, *n* (%)		733 (46.9)	604 (45.3)	129 (55.8)	0.003
Systolic blood pressure, mm Hg		126.9 ± 21.3	126.9 ± 20.6	127.1 ± 24.8	0.899
Diastolic blood pressure, mm Hg		82.1 ± 14.5	77.6 ± 13.8	74.7 ± 15.2	0.004
Heart rate, bpm		82.1 ± 14.5	81.4 ± 13.9	86.0 ± 17.4	<0.001
Left ventricular ejection fraction, %		49.1 ± 9.7	49.3 ± 9.5	47.5 ± 10.3	0.009
Killip classification, *n* (%)					<0.001
	I	1111 (71.1)	980 (73.6)	131 (56.7)	
	II	273 (17.5)	235 (17.6)	38 (16.5)	
	III	134 (8.6)	93 (7.0)	41 (17.7)	
	IV	45 (2.9)	24 (1.8)	21 (9.1)	
**Risk factors, *n* (%)**					
Hypertension		727 (46.5)	612 (45.9)	115 (49.8)	0.285
Diabetes mellitus		692(44.3)	574 (43.1)	118 (51.1)	0.024
PCI history		282 (18.0)	222 (16.7)	60 (26.0)	<0.001
CABG		267 (17.1)	224 (16.8)	43 (18.6)	0.508
Chronic HF		194 (12.4)	146 (11.0)	48 (20.8)	<0.001
Hyperlipidemia		399 (25.5)	334 (25.1)	65 (28.1)	0.327
Smoking					<0.001
	No	790 (50.5)	683 (51.3)	107 (46.3)	
	Former	439 (28.1)	393 (29.5)	46 (19.9)	
	Current	334 (21.4)	256 (19.2)	78 (33.4)	
**Laboratory findings**					
Glucose, mg/dL		169 ± 88	165 ± 85	193 ± 100	<0.001
Urea, mg/dL		38.1 ± 17.3	36.9 ± 15.7	44.8 ± 23.3	<0.001
Creatinine, mg/dL		0.93 ± 0.50	0.90 ± 0.48	1.08 ± 0.59	<0.001
e-GFR, mL/min/1.73 m^2^		85.6 ± 23.1	87.3 ± 21.5	76.3 ± 29.4	<0.001
hs-CRP, mg/L		5.7 (2.5–12.7)	5.4 (2.4–11.7)	8.1 (3.8–18.3)	<0.001
Albumin, g/dL		38.6 ± 4.3	39.1 ± 4.0	35.4 ± 4.9	<0.001
ALT, IU/L		22 (16–31)	22 (16–31)	22 (16–35)	0.652
AST, IU/L		30 (22–48)	30 (22–48)	30 (22–46)	0.845
Total Bilirubin, μmol/L		9.91 ± 5.89	9.46 ± 5.66	12.56 ± 6.49	<0.001
Sodium, mmol/L		137.6 ± 3.0	137.7 ± 2.9	137.1 ± 3.5	0.003
Potassium, mEq/L		4.3 ± 0.5	4.3 ± 0.5	4.4 ± 0.5	0.010
White blood cell, ×10^3^/μL		10.8 ± 3.3	10.7 ± 3.2	10.9 ± 3.7	0.305
Hemoglobin, g/dL		13.6 ± 1.9	13.7 ± 1.8	12.8 ± 2.1	<0.001
Platelets, ×10^3^/μL		260 ± 75	260 ± 72	265 ± 85	0.341
hs-TnI, ng/L		857 (225–2798)	681 (193–2567)	2552 (720–7071)	<0.001
CK-MB, ng/mL		12.9 (6.1–25.8)	13.0 (6.4–25.8)	12.3 (5.2–28.2)	0.275
HDL-C, mg/dL		42 ± 12	42 ± 12	42 ± 11	0.948
LDL-C, mg/dL		129 ± 34	130 ± 33	120 ± 35	<0.001
Total-C, mg/dL		193 ± 46	194 ± 45	184 ± 48	0.003
Triglycerides, mg/dL		148 (102–206)	105 (102–207)	143 (99–203)	0.556
**Medications taken at the time of admission, *n* (%)**					
Acetylsalicylic acid		194 (12.4)	177 (13.3)	17 (7.4)	0.010
ACEi or ARB		643 (41.1)	527 (39.6)	116 (50.2)	0.002
Beta-blockers		192 (12.3)	175 (13.1)	17 (7.4)	0.012
Statins		158 (10.1)	117 (8.8)	41 (17.7)	<0.001
Oral anticoagulants		42 (2.7)	40 (3.0)	2 (0.9)	0.102
GRACE Score		137 ± 30	137.5 ± 29	137 ± 32	0.916
ALBI Score		−2.66 ± 0.40	−2.72 ± 0.37	−2.31 ± 0.44	<0.001

Values are presented as *n* (%), median (interquartile range [IQR]_25–75_), or mean ± standard deviation (SD). * A *p*-value of <0.05 was considered statistically significant. Abbreviations: ACEi, angiotensin-converting-enzyme inhibitors; ALT, alanine transaminase; ARB, angiotensin receptor blockers; AST, aspartate aminotransferase; CABG, coronary artery bypass surgery; e-GFR, estimated glomerular filtration rate; HDL-C, high-density lipoprotein cholesterol; HF, heart failure; hs-CRP, high sensitivity C-reactive protein; hs-TnI, high-sensitivity troponin I; LDL-C, low-density lipoprotein cholesterol; Total-C, total cholesterol; WBC, white blood cell.

**Table 2:** Angiographic and procedural features.

Variable		Pooled (*n* = 1563)	No-Reflow (−) (*n* = 1332)	No-Reflow (+) (*n* = 231)	*p*-Value *
Culprit vessel, *n* (%)					0.255
	LM	38 (2.4)	31 (2.3)	7 (3.0)	
	LAD	609 (39.0)	523 (39.3)	86 (37.2)	
	LCx	323 (20.7)	264 (19.8)	59 (25.5)	
	RCA	428 (27.4)	374 (28.1)	54 (23.4)	
	Graft	165 (10.6)	140 (10.5)	25 (10.8)	
Thrombus burden, *n* (%)					0.425
	Low (0–1–2–3)	925 (59.2)	794 (59.6)	131 (56.7)	
	High (4–5)	638 (40.6)	538 (40.4)	100 (43.3)	
GP IIb/IIIa inhibitor infusion, *n* (%)		249 (15.9)	119 (8.9)	130 (56.3)	<0.001
In-hospital shock, *n* (%)		88 (5.6)	62 (4.7)	26 (11.3)	<0.001
Malignant arrhythmia, *n* (%)		114 (7.3)	66 (5.0)	48 (20.8)	<0.001
Pre-dilatation, *n* (%)		1122 (71.8)	989 (74.2)	133 (57.6)	<0.001
Multi-vessel disease, *n* (%)		312 (20.0)	252 (18.9)	60 (26.0)	0.016
In-hospital mortality, *n* (%)		87 (5.6)	58 (4.4)	29 (12.6)	<0.001
High-dose statin, *n* (%)		1471 (94.1)	1247 (93.6)	224 (97.0)	0.048
Antiplatelet therapy, *n* (%)					0.053
	Clopidogrel	235 (15.0)	208(15.6)	27 (11.7)	
	Ticagrelor	1264 (80.9)	1075 (80.7)	189 (81.9)	
	Prasugrel	64 (4.1)	49 (3.7)	15 (6.5)	

Values are presented as numbers (*n*) and percentages (%). * A *p*-value of <0.05 was considered statistically significant. Abbreviations: LM, left main; LAD, left anterior descending artery; LCx, left circumflex artery; RCA, right coronary artery; GP, glycoprotein.

**Table 3:** Binary logistic regression analysis of the no-reflow phenomenon in NST-ACS patients undergoing PCI.

Variable	Co-Variate Effect			Fitted Model			ALBI Model		
	OR	95% CI	*p*-Value *	OR	95% CI	*p*-Value *	OR	95% CI	*p*-Value *
hs-cTnI	1.00	1.00–1.00	<0.001	1.00	1.00–1.00	<0.001	1.00	1.00–1.00	<0.001
e-GFR	0.98	0.98–0.98	<0.001	0.99	0.98–1.00	0.053	0.99	0.99–1.00	0.236
Pre-dilatation, *yes*	0.47	0.35–0.63	<0.001	0.24	0.14–0.40	<0.001	0.24	0.14–0.40	<0.001
Hemoglobin	0.78	0.72–0.84	<0.001	0.82	0.75–0.90	<0.001	0.84	0.77–0.93	<0.001
Glucose	1.00	1.00–1.01	<0.001	1.00	1.00–1.00	0.056	1.00	1.00–1.00	0.078
CK-MB	0.99	0.99–1.00	0.576	0.99	0.98–0.99	0.011	0.99	0.99–1.00	0.043
Malignant arrhythmia, *yes*	0.96	3.31–7.40	<0.001	4.58	2.92–7.17	<0.001	4.61	2.81–7.56	<0.001
Statins use at admission, *yes*	2.24	1.52–3.30	<0.001	3.45	2.18–5.43	<0.001	4.10	2.51–6.71	<0.001
Sodium	0.93	0.90–0.98	0.004	0.97	0.92–1.03	0.342	0.95	0.90–1.01	0.101
Triglycerides	1.00	0.99–1.00	0.918	1.00	0.99–1.00	0.549	1.00	1.00–1.00	0.238
LVEF	0.98	0.97–1.00	0.009	1.00	0.98–1.02	0.872	1.00	0.98–1.03	0.480
GRACE score, *continuous*	1.00	0.99–1.00	0.916	0.99	0.99–1.01	0.835	1.00	0.99–1.01	0.303
Thrombus burden, *from* 0 *to* 5, *reference:* 0	-	-	<0.001 ^+^	-	-	<0.001 ^+^	-	-	0.128 ^+^
ALBI score, *continuous*	10.28	7.16–14.79	<0.001	-	-	-	12.10	7.75–18.89	<0.001

^+^ trend analysis *p*-value. * A *p*-value of <0.05 was considered statistically significant. Abbreviations: CI, confidence interval; e-GFR, estimated glomerular filtration rate; hs-cTnI, high-sensitivity cardiac troponin I; LVEF, left ventricular ejection fraction; OR, odds ratio.

**Table 4:** Discrimination and reclassification analyses to estimate compared with the ALBI score for no-reflow phenomenon in non-ST elevation acute coronary syndrome patients undergoing PCI.

	Discrimination and Reclassification							
	Goodness of Fit		Net Reclassification Improvement		Integrated Discrimination Index		Median Improvement	
	C-Index (95% CI)	*p*-Value *	NRI Index (95% CI)	*p*-Value *	IDI Index (95% CI)	*p*-Value *	Median Improvement Index (95% CI)	*p*-Value *
Fitted model ^§^	0.799 (0.764–0.829)	<0.001	*ref*	*-*	*ref*	*-*	*-*	*-*
ALBI model ^‖^	0.850 (0.831–0.884)	<0.001	0.145 (0.102–0.189)	0.002	0.111 (0.085–0.136)	<0.001	0.105 (0.082–0.146)	<0.001

^§^ Note that the fitted model consists of high-sensitive cardiac troponin I, estimated glomerular filtration rate, hemoglobin, CK-MB, pre-dilatation before stenting, GRACE score, malignant arrhythmia, high thrombus burden, sodium, triglyceride, and left ventricular ejection fraction. ^‖^ Note that the ALBI model consists of ALBI score and fitted model variables. * A *p*-value of <0.05 was considered statistically significant. Abbreviations: CI, confidence interval; IDI, integrated discrimination improvement; NRI, net reclassification index.

## Data Availability

Data are available on request due to privacy and ethical restrictions.
